# A systematic review of the cost-effectiveness of maternity models of care

**DOI:** 10.1186/s12884-023-06180-6

**Published:** 2023-12-13

**Authors:** Elizabeth Martin, Bassel Ayoub, Yvette D. Miller

**Affiliations:** 1 Wesley Research Institute, Auchenflower, Qld Australia; 2 Mater Research Institute – University of Queensland, South Brisbane, Qld Australia; 3 School of Public Health and Social Work, Faculty of Health, Centre for Healthcare Transformation, Queensland University of Technology, Brisbane, Qld Australia; 4 School of Public Health and Social Work, Faculty of Health, Queensland University of Technology, Brisbane, Qld Australia

**Keywords:** Cost-effectiveness, Maternity models of care, Markov, Health economics, Midwifery, Doula

## Abstract

**Objectives:**

In this systematic review, we aimed to identify the full extent of cost-effectiveness evidence available for evaluating alternative Maternity Models of Care (MMC) and to summarize findings narratively.

**Methods:**

Articles that included a decision tree or state-based (Markov) model to explore the cost-effectiveness of an MMC, and at least one comparator MMC, were identified from a systematic literature review. The MEDLINE, Embase, Web of Science, CINAHL and Google Scholar databases were searched for papers published in English, Arabic, and French. A narrative synthesis was conducted to analyse results.

**Results:**

Three studies were included; all using cost-effectiveness decision tree models with data sourced from a combination of trials, databases, and the literature. Study quality was fair to poor. Each study compared midwife-led or doula-assisted care to obstetrician- or physician-led care. The findings from these studies indicate that midwife and doula led MMCs may provide value.

**Conclusion:**

The findings of these studies indicate weak evidence that midwife and doula models of care may be a cost-effective or cost-saving alternative to standard care. However, the poor quality of evidence, lack of standardised MMC classifications, and the dearth of research conducted in this area are barriers to conclusive evaluation and highlight the need for more research incorporating appropriate models and population diversity.

**Supplementary Information:**

The online version contains supplementary material available at 10.1186/s12884-023-06180-6.

## Highlights

• No conclusions could be drawn regarding the cost-effectiveness of individual MMCs due to the limited and low-quality studies eligible for inclusion in this review.

• Very few studies exist in the literature that use modelling to assess the cost-effectiveness of MMCs. This paucity of evidence hinders the determination of the best value maternity services and may lead to inappropriate policy and funding.

**Supplementary Information:**

The online version contains supplementary material available at 10.1186/s12884-023-06180-6.

## Background

Recognition of women’s diverse needs, circumstances and preferences has resulted in large investments internationally to expand the range and accessibility of models of maternity care (MMC) [1–3]. MMCs are care pathways pregnant women engage in for their maternity care, guiding the level and type of care provided to a woman during pregnancy, birth and the postpartum period [4]. At least 10 distinct MMCs are available internationally in high income countries, and these can be categorized into five broad groups: Standard Care delivered by a large team of obstetricians and midwives; General Practitioner (GP) or Family Physician Shared Care with support from obstetricians as required; Midwife-led Continuity Care with support from obstetricians as required; Private Obstetric-led Continuity Care; and Private Midwife-led Continuity Care, each organised individually by the pregnant woman [5–7]. Features of each group are described in Table 1, outlining who leads the care and usual location of care. While broad categories exist, and efforts have been made to develop a standardised classification system [4], varied and inconsistent terminologies and definitions around MMCs remain an impediment to adequate evaluation of available MMCs [7]. Funding of MMCs varies internationally, with universal health care or public health insurance funding many MMCs in countries including Australia, the United Kingdom and Netherlands [8, 9]. Private health insurance, supplemented by Affordable Care Act funding and out-of-pocket fees, finances MMCs in the United States [10, 11].

**Table 1:** International maternity model of care categories and features

Maternity Model of Care Category	Main Care Provider	Location of Pregnancy Check-ups	Location of Birth
Standard Care	Rostered hospital doctors and midwives	Hospital or community clinic	Hospital
General Practitioner (GP)/Family Physician Shared Care	Community maternity service provider (GP and/or midwife)	General Practitioner clinic or hospital	Hospital
Midwife-led Continuity Care	Health service midwife or small team of midwives^a^	Hospital, community clinic, birth centre (co-located or stand-alone), or patient’s home	Hospital, birth centre, or patient’s home
Obstetric Continuity Care	A private obstetrician of choice and rostered private hospital midwives	Hospital or obstetrician’s clinic	Hospital
Private Midwife-led Continuity Care	A private midwife of choice	Patient’s home or midwife’s clinic	Hospital, birth centre or patient's home

^a^Often called team, caseload/group practice

A major challenge for health systems is that it is not sustainable to continue to expand access to a wider range of MMCs. Ill-informed expansion would create inefficiencies in both a free or government regulated market. Inefficiencies would arise because of an excess supply of an inappropriate mix of services that do not meet the demand for different MMCs. This situation may result in ‘too little care, too late’ or ‘too much, too soon’; and alongside the high costs of establishing maternity care, would be inefficient for the health system [12, 13]. However, there is justification for selective expansion of MMCs that are cost-effective. Funding of MMCs that improve health outcomes and/or costs relative to alternatives is likely to free resources for other types of maternity services that are justified on equity and access grounds, creating a fair health system. It is therefore critical that decision makers have a thorough understanding of the cost-effectiveness of MMCs.

Existing evidence for the costs alone, or cost-effectiveness of MMCs is limited especially for team and caseload midwife-led continuity MMCs [14, 15]. Studies that have examined both costs and health outcomes of various MMCs have generally not used economic modelling methods which establish a generalizable framework for future research and service evaluations. The available evidence on costs and outcomes of midwife-led continuity models versus other MMCs reported in randomized controlled trials has been well synthesized in a Cochrane review [16]. Four studies included in this Cochrane review examined costs alongside maternal and neonatal clinical outcomes [17–20] but did not provide a combined measure of costs and health outcomes such as an incremental cost-effectiveness ratio, nor use cost-effectiveness modelling methods. Doing so would have better-informed decision makers as to the real-world economic costs of a full clinical pathway associated with different MMCs, rather than the not generalizable conclusion of the Cochrane review that midwife-led continuity MMCs may be cost-saving [16]. Model-based economic evaluations were also found to be rare by authors of an Australian review that focused on midwife-led continuity MMCs for women with high-risk pregnancies [21]; and uncommon in a systematic review of the cost-effectiveness of midwife-led care in the United Kingdom [13]. Another limitation of existing literature is studies evaluating the cost-effectiveness of place of birth being perceived as equivalent to an evaluation of MMCs [22–24]. While place of birth is often unique to individual MMCs, the available evaluations do not examine the full pregnancy and postpartum continuum where approaches to care, and therefore adverse events and costs, may differ between MMCs. It is important to evaluate the full range of MMCs available internationally, rather than focusing on one aspect such as place of birth or one profession.

While there is clear evidence that midwife-led continuity care MMCs result in better short-term clinical outcomes for low-risk mothers and neonates, such as fewer caesarean sections and admissions to neonatal intensive care units [20, 25, 26], decision makers can only design an efficient mix of MMCs when all options are evaluated and produce a useful measure of benefit that combines both costs and outcomes into an incremental cost-effectiveness ratio or equivalent. We suspect there is little useful and relevant evidence for the cost-effectiveness of MMCs that can inform maternity care reform internationally. In the ‘place of birth evaluation’ conducted by Henderson et al., the authors acknowledge the dearth of model-based economic evaluations examining MMCs more widely [22] which is supported by the more recent Australian review [21].

### Aims

The aims of this systematic review were to determine the extent of cost-effectiveness evidence available for evaluating alternative MMCs and to narratively summarize findings for their comparative cost-effectiveness. We intended to identify gaps in knowledge that may prohibit cost-effectiveness analysis being used to support maternity services in their allocation of resources to MMCs internationally.

## Methods

### Registration and reporting

The protocol for this systematic review has been registered with PROSPERO: CRD42021223334. Our reporting is guided by the 2020 PRISMA statement [27].

### Search strategy

The search strategy was developed collaboratively, with consensus from the review team and input from an experienced librarian. We performed thorough and systematic searches in Embase, MEDLINE, Web of Science, and Google Scholar (from which only the first 200 references were included). These databases were expected to capture 98.3% of relevant studies [28]. For completeness, we also searched the Cumulative Index of Nursing and Allied Health Literature (CINAHL Complete via EBSCOhost) [28]. Additionally, the reference lists of studies included in full-text screening and relevant systematic reviews were hand-searched.

The search strategy included keywords and subject headings related to models of maternity care (e.g., private obstetric care, birth centre care, midwifery group practice) and economic evaluation research (e.g., cost-effectiveness, cost-benefit, cost-utility). Searches were adapted for appropriate use in each database.

The search was restricted to papers published in English, Arabic and French. Studies published from 01/01/2000 until 23/11/2020 were sought to ensure both a broad search and more recent costing estimates amongst included studies. Searches were repeated across all databases on 30/12/2022 to include the years 2021 and 2022 before the final analysis, and newly published studies considered for inclusion. The full search strategy will be available on PROSPERO upon publication of this review.

### Eligibility criteria

To be included in this review, papers had to report on findings from cost-effectiveness modelling studies that used a decision tree or state-based (Markov) model to explore the cost-effectiveness of an MMC and at least one comparative MMC. We only sought modelling studies because they are excellent at comparing all the relevant alternatives that a decision maker is considering, simplifying reality where a ‘real life’ randomized-controlled trial cannot be replicated, such as randomly allocating women to different MMCs [29]. Models also have the flexibility to use multiple sources of data, ensuring the best available data informs decision making. Measuring single clinical outcomes in trial-based economic evaluations are only justifiable where there is good reason to believe that the change will not also have long term effects on quality of life and decision makers are not interested in other relevant intervention outcomes, which is not appropriate for maternity care. Models also have an advantage over clinical trial-based economic evaluations in that they can be easily adjusted to other settings such as regular practice and other geographical locations [29].

Studies which solely focused on specific interventions during pregnancy were excluded. Location or ward-based studies that looked at the impact of midwife-led versus obstetrician-led wards or similar were also excluded as there was a consensus between the reviewers that admission to either type of ward does not necessarily indicate the women’s affiliation with either model of care; a woman could have been following a midwife-led model but assigned to an obstetrician-led ward and their outcomes may not necessarily be a reflection of obstetrician-led care in a ward. Women in the included studies needed to have remained in a single MMC across the continuum of pregnancy, birth and the postpartum period. If other specialists were involved in care and the lead clinician or model of care structure continued, these studies were eligible for inclusion. If it was not reported that women moved between MMCs, either in reality or hypothetically in the cost-effectiveness model, then the studies were included.

### Study selection

Recalled citations were exported to EndNote. Duplicates were removed. Two independent reviewers EM and BA screened screen titles and abstracts against eligibility criteria. Conflicts were resolved through discussion. EM and BA then both independently screened all full-text papers and reached a consensus on the included studies.

### Outcome measures

The primary outcome was an incremental cost-effectiveness ratio representing the change in costs and change in health outcomes observed when comparing one MMC to another. As a secondary outcome, we were also interested in whether model uncertainty had been quantified.

### Data extraction analysis

Data extraction was conducted using Excel. Extracted information included the MMCs evaluated, the cost-effectiveness modelling approach used with relevant parameters and assumptions, reported incremental cost-effectiveness ratio, the selected cost-effectiveness threshold, the reported costs and health outcomes for each evaluated MMC, and sensitivity analysis results. These parameters were decided upon following the Consolidated Health Economic Evaluation Reporting Standards (CHEERS) statement [30], and the authors’ expertise.

### Quality assessment

Quality of included studies was assessed using the Joanna Briggs Institute (JBI) Checklist for Economic Evaluations [31]. Included studies were categorised as ‘good’, ‘fair’ and ‘poor’ using the criteria described in the protocol. Since this was a narrative synthesis review and no papers were to be excluded based on their methodological quality, the authors deemed it appropriate to have one reviewer (BA) appraise the studies with thorough cross checking from EM, and conflicts resolved through discussion.

### Narrative data synthesis

A narrative synthesis was conducted, rather than a meta-analysis because of: the range of MMCs delivered internationally and therefore evaluated; the variation in potential primary outcome measures; and the expected small number of good quality economic evaluations. In the synthesis, we describe the included studies and report the cost-effectiveness and any sensitivity analysis results.

## Results

**Fig. 1:**
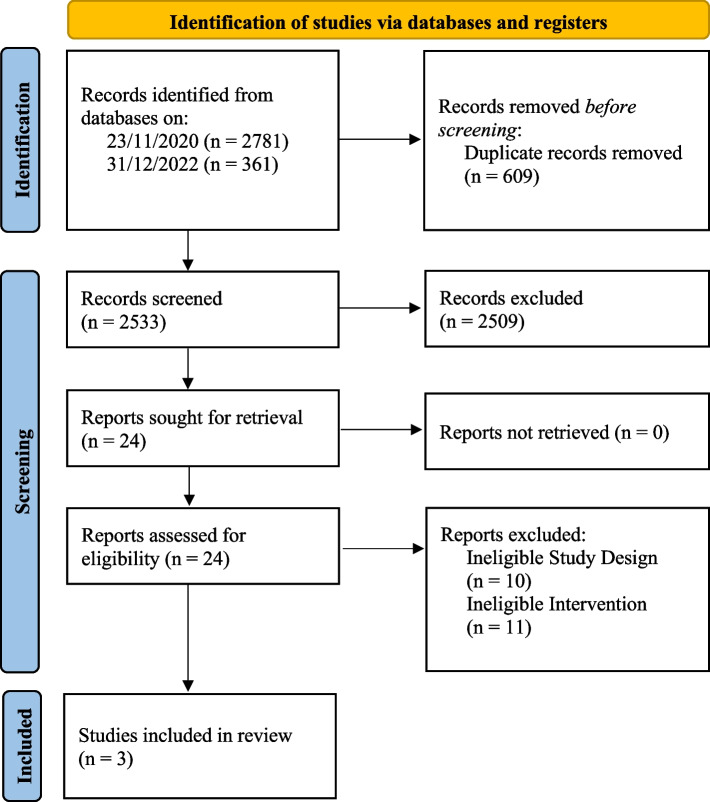
PRISMA Flow Diagram

We identified 3142 potential studies for inclusion, of which 2533 remained after the removal of duplicates. We excluded 2509 studies during title and abstract screening, and the remaining 24 papers underwent full-text screening. From these, three studies were included for data extraction and synthesis (see Fig. 1). All three studies were cost-effectiveness decision tree models, with data sourced from a combination of trials, databases and the literature. Excluded studies following abstract screening and full-text assessment for eligibility with reasons are in an additional file (see Additional file 1).

The quality of the included studies ranged from poor [32] to fair [33, 34], with none being rated as good. The poor-quality study did not report costs and health outcomes clearly, nor were costs converted to a single consistent year [32]. They also failed to report how potential confounding factors were considered during analysis, such as maternal health and socioeconomic factors, that may have greatly impacted the external validity and subsequent conclusions of the study. The key limitation of the two studies assessed as fair was that they did not make relevant assumptions that are key for decision making and policy. For example, one study assumed a single cost for neonatal intensive care, whereby in reality it would depend on length of stay [33]. The primary outcome of this study – neonatal intensive care avoided – does not represent the quality of life of the parents. Such an outcome also potentially undervalues the baby’s life while admitted, as the outcome is binary and does not represent a family’s journey through intensive care. The other fair study did not distinguish between emergency and elective caesarean section outcomes which incur very different maternal quality of life outcomes [34]. Quality assessment ratings for the three studies are available in Table 2.

**Table 2:** Summary of included studies’ features and results

Authors (publication year)	Critical appraisal score (rating)	Country	MMC evaluated	Population	Model choice	Perspective	Time horizon	Primary health outcome	Main conclusion
Kozhimannil et al. (2016) [34]	8/11 (Fair)	United States	Women cared for by midwife vs women cared for by family physicians	Low risk pregnancies	Decision tree	Payer	Antepartum up to birth	Preterm birth (<37wks) averted	Midwife-led is cost-saving
Koto et al. (2019) [33]	8/11 (Fair)	Canada	Standard care with doula support vs standard care	Low risk pregnancies	Decision tree	Payer	Antepartum, intrapartum, and up to six weeks postpartum	NICU admission avoided	Additional doula support is cost-effective
Attanasio et al. (2020) [32]	5.5/11 (Poor)	United States	Midwife-led care vs obstetrician led care	Low risk pregnancies	Decision tree	Payer	Antepartum up to birth	Obstetric procedures during child- birth	Midwife-led is cost-saving

The three studies were conducted in Canada [33], and the United States [32, 34] (Table 2). In the Canadian study [33], the cost-effectiveness of a family physician versus midwife-led MMC was evaluated between 2013 and 2017. Decision tree modelling was conducted from the perspective of the province (state) of Nova Scotia, as the government was evaluating the new midwife led MMC for low-risk pregnancies. Avoiding Neonatal Intensive Care Unit (NICU) admission was the primary health outcome, while costs were estimated from standard public health insurance costs associated with maternity care activity logged in hospital administrative databases. The incremental cost-effectiveness ratio of midwife-led care was C$ 27,502 (US$22,090) per NICU case avoided. The authors used a cost-effectiveness threshold of C$50,000 (US$40,160) per NICU cases avoided and the midwife-led care was therefore deemed cost-effective, but not cost-saving.

In one of the studies conducted in the United States [32], obstetrician-led care was compared to midwife-led care for low-risk women between 2011 and 2012. Decision tree modelling was conducted from the perspective of health funders including the public Medicaid program and private health insurers. The primary health outcome was obstetric procedures during birth such as epidural analgesia, labour induction, caesarean birth and episiotomy. The best available health data was synthesised for the decision tree model: a combination of data from a national cross-sectional dataset of women and a systematic review [16]. Costs were sourced from reports of private and public care costs in the United States. While no incremental cost-effectiveness ratio was reported, the authors concluded that a shift from obstetrician led care to midwife led care could be cost saving in the United States.

The second study conducted in the United States [34] was a decision tree modelling evaluation of standard maternity care compared to standard care plus doula support in the upper Midwest between 2010 and 2014. A doula is a non-medical companion who can provide support before, during and after birth. The aim of this study was to model the potential cost-effectiveness of Medicaid-funded doula services. Standard maternity care was not defined in the included study, but we assumed was obstetrician-led with the women being cared for by a variety of different obstetricians and midwives during the care continuum, with delivery in a Medicaid-funded hospital. This model is similar to standard maternity Medicaid-funded care described elsewhere [11]. While doula services are not internationally regarded as a type of MMC, this study was included as a potentially novel additional MMC which is different again to midwife-led continuity of care. The primary health outcome was preterm birth (< 37 weeks) averted and health data for the model was synthesised from multiple sources: a national survey; maternity care activity database; government pre-term birth data; and data from women and the doulas who participated in the doula trial. Costs of care were sourced from Medicaid government reports. An incremental cost-effectiveness ratio was not reported, however a scatterplot in the included study suggested that standard care with a Medicaid-funded doula would be cost saving based on deterministic modelling.

All three included studies used probabilistic sensitivity analysis to quantify uncertainty in the models. Midwife led MMCs in Canada had an 83% probability of being cost-effective. In this study, scenarios of increasing costs of care, and sub-group analyses across urban and rural areas in Canada were also assessed. The cost-effectiveness results remained below the set cost-effectiveness threshold in these scenarios [33]. In the authors’ sensitivity analysis for the United States study comparing midwife led and obstetric led care [32], 95% prediction intervals were reported for both outcomes of costs and obstetric procedures used in the decision tree model. Costs had narrow prediction intervals: C$28,457-C$30,936 for obstetrician led care, and C$25,426-C$29,108 for midwife led care. Wider prediction intervals were reported for health outcomes of preterm births, planned caesarean section, epidural and episiotomy [32]. In the scenario analysis, the authors tested two different increases in the volume of births cared for in a midwife led MMC: a 10 percentage-point increase and an increase from 8.9 to 40%. Costs savings increased for each scenario. An increase in the proportion of midwife led care from 8.9 to 40% would yield annual cost savings of US$539 million for public funders, and a similar shift toward midwife led care would save the private health sector US$1.35 billion. Adding doula support to standard care in the second United States study had a 73.3% probability of being cost-saving, as reported in the sensitivity analysis [34].

The drivers of cost-effectiveness proposed across all three included studies were reduced birth interventions [33], specifically epidural, episiotomy [32] and caesarean section [32, 34], and fewer preterm births [32, 34].

## Discussion

Through this systematic review, we identified three studies that examined cost-effectiveness of different MMCs in low-risk pregnancies using decision tree modelling. Each study compared midwife-led or doula-assisted care to obstetrician- or physician-led care. All studies concluded that midwife and doula-assisted models of care would be cost-effective or cost-saving. Costs were estimated from public reports of healthcare costs and existing literature, and often generalised disparate treatment types into single cost estimations. In all studies, low risk pregnancies were treated as a homogenous group, which may impede findings of true cost savings in particular population segments. Overall, the quality of included studies was poor to fair which impacts the interpretation of the studies’ results for other settings.

All studies had significant methodological limitations. One study [32] did not record any medical or demographic data of participants. Along with the self-selection bias inherent in observational research of this type of medical care, potential confounders may greatly influence health and cost outcomes. The other two studies attempted to control for confounders, but the findings show that the midwife-led cohorts did have lower rates of risk factors for poor birth outcomes such as obesity, smoking, hypertension and diabetes [33, 34].

The findings of these studies indicate that there is weak evidence that midwife and doula models of care may be a cost-effective or cost-saving alternative to standard care. However, the low quality of evidence, lack of health and demographic data, self-selecting bias, and inappropriate cost measurement procedures and assumptions, mean that further research will need to be conducted in order to determine the true economic impacts of differential models of care and identify the patient groups for which these models may be most suitable.

Modelling studies for evaluating the cost-effectiveness of alternative MMCs were rare. This may be due to the difficulties associated with valuing temporary health states such as pregnancy [35]. The value of health states for long term chronic conditions are well studied [36–42]. However temporary health states where the disutility is experienced for 1 year or less with a usual return to normal health, although we acknowledge that some women never return to normal health in the post partum, is not well researched [35]. It is argued [43] that estimating the value people place on states of health using time trade-off or standard gamble methods is not appropriate because of their comparison of the health state in question to death, which may be too extreme a comparison. For example, population data used to value states of health represented by EQ-5D-5L responses and using conventional time-trade off methods [43] are therefore likely to be an inaccurate value of temporary health states such as those experienced during pregnancy. Adapted methods for valuing temporary health states have been proposed [35] and we recommend further study in this area for economic evaluations of maternity services.

There is also a general paucity of long-term data for maternity outcomes, such as breastfeeding outcomes and infant atopy. Few modelling studies for the economic evaluation of maternity services may also be explained by the lack of this data and challenges of including infant health outcomes in models [44]. The clinical pathway of one type of person is usually represented by a model structure, and no guideline has been established for incorporating the health outcomes of both women and infants in a single model. As the mother-baby dyad is such a critical aspect of maternity care, the availability of long-term data that can populate a model examining both women’s and infants’ health outcomes and costs following engagement with maternity services is important to pursue.

### Strengths and limitations

The systematic review had a number of strengths, including adherence to well-regarded reporting and quality assessment tools [30, 31]. We also created a thorough and focussed search strategy with a well-defined methodology for the purposes of identifying whether a research gap exists in terms of available cost-effectiveness evidence to inform decisions to expand MMCs. The authors were able to further increase the validity of the findings by widening the breadth of the search to include Arabic and French manuscripts.

The main limitation, or barrier, to this review is the lack of standardised classifications for models of maternity care. In 2022, 890 different MMCs were reported in Australia alone [45]. While standardised classification systems have been developed, heterogeneity within models invalidates many of these attempts at standardisation [46]. Other limitations of the review result from the lack of diversity in the MMCs being evaluated in included studies. While there are a large number of maternity care pathways to choose from, studies only included midwife-led care and doula-supported care, comparing these to obstetrician or family physician-led care. Studies also lacked geographic diversity, with two conducted in the United States of America, and one in Canada. Therefore, the findings can only be generalised to these two nations, with generalisability not possible between these two even. Data relating to the types and costs of MMCs in countries with different culture and health system structure to North America, and the economic consequences of prioritising specific models, is yet to be identified or evaluated.

## Conclusions

In this systematic review we identified three studies that used decision tree modelling to determine the cost-effectiveness of alternative MMCs. Few studies use appropriate and rigorous cost-effectiveness modelling to enhance the strength of evidence evaluating MMCs. Findings from the limited number of studies available for this review consistently indicate that midwife- and doula-led MMCs provide value. More confident conclusions were prevented by low study quality, and limited generalizability of results. Further research could better reflect the complexity and diversity of the MMC service-delivery landscape and the range of outcomes across the mother-baby dyad. Lack of standardised nomenclature for MMCs is an additional impediment to building evidence in this area. Future cost-effectiveness studies evaluating MMCs should explore new methods that measure mother-baby dyad outcomes, and account for the diversity of the MMCs internationally.

### Supplementary Information

**Additional file 1: Appendix 1. **Reasons for exclusion.

## Data Availability

The datasets used and/or analysed during the current study are available from the corresponding author on reasonable request.
