# Supplementary material for: A systematic review of the cost-effectiveness of maternity models of care
Source: BMC Pregnancy Childbirth. 2023 Dec 13;23:859. doi: 10.1186/s12884-023-06180-6 (PMC10717830; doi:10.1186/s12884-023-06180-6)
Supplement: Supplementary file 1 — Additional file 1: Appendix 1. Reasons for exclusion. [file 12884_2023_6180_MOESM1_ESM.docx]

**Appendix 1:** Reasons for exclusion

| Full text papers | Reason for exclusion |
| --- | --- |
| Broughton et al. 2011 | Ineligible intervention - only clinical interventions evaluated, not models of care. Focuses on third stage labour |
| Stone et al. 2000 | Ineligible intervention - no health outcomes evaluated, only clinical resources/technical procedures. |
| Adam et al. 2005 | Ineligible intervention - Only clinical interventions evaluated, not models of care |
| Walters et al. 2015 | Ineligible intervention - focuses solely on a single profession (the caring practitioner at birth) |
| Montfort et al. 2020 | Ineligible intervention - Only clinical interventions evaluated, not models of care |
| Villar et al. 2001 | Ineligible study design - no cost effectiveness modelling - minimal CE analysis with no incremental health outcomes reported |
| Borghi et al. 2005 | Ineligible intervention - women’s group intervention applicable to all models of care but not a model of care on its own |
| Ryan et al. 2013 | Ineligible study design - literature review |
| Bernitz et al. 2012 | Ineligible intervention - Place of birth evaluated, not a model of care |
| Isaline et al. 2019 | Ineligible study design – no cost effectiveness modelling |
| Blais et al. 2000 | Ineligible study design - pilot project description |
| Daviaud et al. 2017 | Ineligible intervention - models of care not evaluated |
| Buser et al. 2018 | Ineligible intervention - models of care not evaluated |
| Barlow et al., 2007 | Ineligible study design - not a cost-effectiveness modelling study |
| Byrne et al. 2000 | Ineligible study design – no cost effectiveness modelling |
| Friedman et al. 2015 | Ineligible study design - commentary/review |
| Reinharz et al. 2000 | Ineligible study design – no cost effectiveness modelling |
| Bartlett et al. 2014 | Ineligible intervention - models of care not evaluated. Examines scaling up the midwifery and obstetrics workforce |
| Greiner et al. 2019 | Ineligible intervention – whole models of care not evaluated only doula support during birth was evaluated. |
| Dubay et al. 2020 | Ineligible study design - not a cost-effectiveness modelling study |
| Callander et al. 2021 | Ineligible study design – did not use a state based or decision tree model |
